# *Euphorbia humifusa* Willd. ex Schltdl. Mitigates Liver Injury via KEAP1-NFE2L2-Mediated Ferroptosis Regulation: Network Pharmacology and Experimental Validation

**DOI:** 10.3390/vetsci12040350

**Published:** 2025-04-09

**Authors:** Hongxu Du, Kunzhao Yang, Jingyi Yang, Junjie Wan, Yu Pan, Weijie Song, Shuang Xu, Cheng Chen, Jiahui Li

**Affiliations:** 1 Department of Traditional Chinese Veterinary Medicine, College of Veterinary Medicine, Southwest University, Chongqing 402460, China; 2 Institute of Traditional Chinese Veterinary Medicine, Southwest University, Chongqing 402460, China

**Keywords:** *Euphorbia humifusa* Willd. ex Schltdl., hepatic injury, ferroptosis, network pharmacology

## Abstract

Liver injury poses major health risks in livestock, necessitating effective therapeutic interventions. This study elucidates the hepatoprotective mechanisms of *Euphorbia humifusa* Willd. ex Schltdl. (EHW) by integrating network pharmacology, molecular docking, and experimental validation. Using a CCl_4_-induced liver injury model mimicking veterinary clinical scenarios, EHW markedly alleviated hepatic damage, demonstrated by reduced liver index, serum ALT and AST levels, histopathological lesions, iron accumulation, inflammatory cytokines, and ferroptosis-associated gene expression. Network pharmacology identified EHW’s core bioactive components (quercetin, kaempferol, and β-sitosterol) and critical targets (*IL-6*, *STAT3*, *HIF-1α*, *PTGS2*, *NFE2L2*, and *KEAP1*) which were linked to ferroptosis and oxidative stress. Molecular docking revealed robust binding affinities between these compounds and ferroptosis-related proteins. *In vivo* validation confirmed that EHW inhibited *KEAP1*, activated *NFE2L2*-mediated antioxidant defenses (upregulating *SOD1* and *NQO1*), restored iron homeostasis (lowering *TFR1*, elevating *FTH1*), and attenuated phospholipid peroxidation by suppressing *ACSL4* and *ALOX12*. These results indicate that EHW mitigates ferroptosis-driven liver injury via KEAP1-NFE2L2 signaling to restore iron homeostasis and reduce oxidative stress, offering a mechanistic foundation for its clinical application in veterinary hepatoprotection.

## Simple Summary

Liver injury, a prevalent disorder in animals with a multifactorial etiology, is critically influenced by ferroptosis—an iron-dependent cell death mechanism. Although *Euphorbia humifusa* Willd. ex Schltdl. (EHW) exhibits pharmacological properties, its role in modulating ferroptosis-associated liver injury remains underexplored. This study evaluated EHW’s therapeutic effects using a CCl_4_-induced murine hepatotoxicity model. EHW administration significantly attenuated liver dysfunction, mitigated ferroptosis, and restored the regulatory pathways linked to iron metabolism and lipid peroxidation. These findings position EHW as a promising therapeutic agent for veterinary liver diseases, primarily through ferroptosis pathway regulation.

## 1. Introduction

The liver is one of the most vital organs in livestock, playing a crucial role in key physiological functions such as synthesis, metabolism, and immunity [1]. Once liver damage occurs, it severely disrupts these essential physiological processes [2]. In veterinary practice, liver injury is commonly caused by factors such as poor feed quality [3], pathogenic microbial infections [4], the ingestion of toxic substances [5], or improper management practices [6]. When liver damage occurs, it can lead to significant harm to the animal’s health. If timely interventions are not implemented, liver injury may rapidly progress, resulting in liver failure and potentially leading to death [7]. Therefore, there is an urgent need to identify effective therapeutic agents to address the common issue of liver damage in veterinary clinical settings.

As a key organ for iron storage and metabolism in the body, maintaining normal iron homeostasis is crucial for the liver to preserve its proper function [8]. Ferroptosis is a recently emerging form of iron-dependent programmed cell death, characterized by the peroxidation of membrane lipids [9]. Various pathways, including redox balance, lipid metabolism, and iron metabolism, can influence the progression of ferroptosis in cells [10]. Research has demonstrated that ferroptosis exerts a dual function in liver diseases. Specifically, in the context of hepatocellular carcinoma, the induction of ferroptosis has been established as a significant strategy to retard tumor progression [11]. However, in the case of non-alcoholic fatty liver disease, alcoholic liver injury, and liver fibrosis, inhibiting ferroptosis has demonstrated beneficial effects in alleviating the condition [12,13,14]. Furthermore, in liver injury studies, Yang et al. [15] found that Maresin 1 could inhibit ferroptosis by activating the *NFE2L2*/*HMOX1*/*GPX4* pathway, thereby reducing liver damage. Similarly, Wei et al. [16] found that Gan Dan Kang, a traditional Chinese medicine (TCM) compound, could alleviate CCL_4_-induced hepatocellular ferroptosis in mice through the activation of the *KEAP1*/*NFE2L2* pathway. In veterinary clinical practice, inhibiting ferroptosis in liver tissue has also been widely recognized as an important approach to mitigating hepatic injury in livestock. For instance, Zhao et al. [17] found that Phlorotannins could alleviate heat-stress-induced liver damage in broiler chickens by reducing hepatic ferroptosis. Similarly, Huang et al. [18] demonstrated that the total flavonoids of *Rhizoma Drynaria* could reverse AFB_1_-induced liver injury in broilers by inhibiting ferroptosis.

*Euphorbia humifusa* Willd. ex Schltdl. (EHW) is an annual herbaceous plant widely distributed in China. As a TCM, it is also known as Dijincao and has been documented in the *Compendium of Materia Medica* (*Ben-Cao-Gang-Mu* in Chinese) since ancient times [19]. According to TCM theory, EHW is considered to have a pungent and neutral nature, with properties that include clearing heat, detoxifying and cooling the blood, and stopping bleeding, as well as promoting diuresis and alleviating jaundice. Modern medical studies have shown that EHW possesses anti-inflammatory [20], antiviral [21], hypoglycemic [22], and anti-tumor effects [23], and its therapeutic efficacy in treating intestinal and hepatic inflammation is widely acknowledged [24]. However, the potential of EHW to alleviate liver damage through the ferroptosis pathway has not yet been reported. Therefore, this study aims to construct a liver injury model in mice using CCL_4_, a classic hepatotoxic substance, and to employ network pharmacology to predict potential targets, followed by experimental validation. The goal is to explore whether EHW can mitigate liver damage through the regulation of ferroptosis and to investigate the underlying mechanisms. The findings from this study may contribute to a deeper understanding of the mechanisms of EHW’s action and provide a theoretical basis for its clinical application.

## 2. Materials and Methods

### 2.1. Reagents and Materials

EHW was purchased from Kangyiyin Biotechnology Co., Ltd. (Bozhou, China). CCL_4_ was obtained from Guangdong Puhui Chemical Technology Co., Ltd. (Shaoguan, China). The reagent kits for aspartate aminotransferase (AST) and alanine aminotransferase (ALT) were purchased from Nanjing Jiancheng Bioengineering Institute (Nanjing, China). Hematoxylin and eosin staining kits were from BKMAM Biology Technology Co., Ltd. (Changde, China), and the Prussian blue staining kit and 2 × Universal Blue SYBR Green qPCR Master Mix were from Wuhan Servicebio Technology Co., Ltd. (Wuhan, China). RNAiso Plus was purchased from TaKaRa Bio Lnc. (Tokyo, Japan), and the All-in-One 5 × RT MasterMix reverse transcription kit was obtained from Applied Biological Materials Inc. (Vancouver, BC, Canada).

### 2.2. Preparation of the Drug

Distilled water was added to a total of 50 g of EHW at a ratio of 15:1. After soaking for 50 min, the mixture was brought to a boil over a high heat and then simmered for 45 min. The liquid was filtered through gauze, and the filtrate was collected. The remaining drug residue was then re-extracted with an equal amount of distilled water, brought to a boil over high heat, and simmered for 45 min. This process was repeated three times. Then, the combined liquid was concentrated using a rotary evaporator to a final concentration of 0.4 g/mL. Finally, a portion of this concentrated liquid was diluted with distilled water to achieve a concentration of 0.2 g/mL. Both concentrations were aliquoted and stored in a refrigerator at 4 °C for later use.

### 2.3. Animal Grouping and Treatment

**Figure 1:**
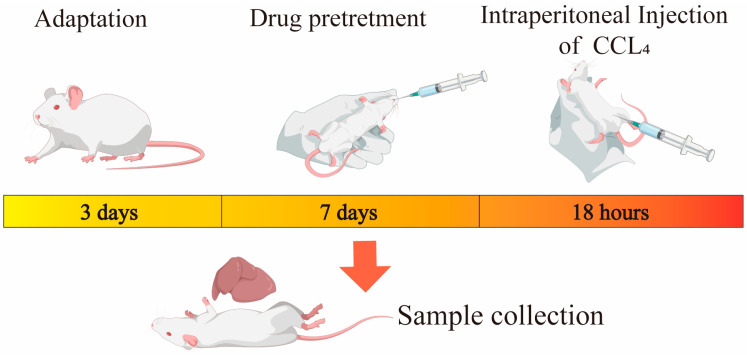
Animal experimental flowchart.

A total of 24 SPF-grade male KM mice (purchased from Southwest Medical University) were randomly assigned into four groups: the control group (CON), model group (CCL_4_), high-dose EHW group (EHW-H), and low-dose EHW group (EHW-L), with 6 mice in each group. After 3 d of acclimatization, all mice were treated with daily oral gavage for 7 consecutive days at a dose of 0.1 mL/10 g of EHW decoction. The high-dose group received EHW at 4 g/kg, and the low-dose group received 2 g/kg. The CON and CCL_4_ groups were given an equivalent volume of sterile water. One hour after the final dose, the mice were injected intraperitoneally with CCL_4_ solution (0.1% CCL_4_ in olive oil) at a dose of 0.1 mL/10 g. Analogously, the CON group received an equivalent volume of olive oil. Eighteen hours later, the mice were gavaged once more and then anesthetized for blood collection from the ocular sinus. Finally, the mice were euthanized via cervical dislocation, and their liver tissues were harvested. Part of the liver was fixed in 4% paraformaldehyde, and another portion was rapidly frozen in liquid nitrogen and stored at −80 °C for long-term preservation. The experimental procedure and treatment flow was created with the BioGDP website (https://biogdp.com/ (accessed on 17 October 2024)) [25], as shown in Figure 1. All animal experiments were conducted in accordance with the guidelines approved by the Ethics Committee of Southwest University for Animal Welfare (Ethics No. LAC2024-1-0346).

### 2.4. Measurement of Liver Organ Index

The liver was carefully removed from the mice, and the surface moisture was gently blotted with bibulous paper. The organs were then precisely weighed using an electronic balance, and the organ index was calculated using the following formula:

Liver Index = Organ Weight (g)/Body Weight (g)

### 2.5. Serum AST and ALT Activity Assays in Mice

Serum samples from mice were used to measure the activities of AST and ALT using the respective test kits, following the instructions provided by the manufacturers. These assays were performed to evaluate the liver function status of the mice.

### 2.6. Hematoxylin and Eosin (HE) Staining

Fresh liver tissue samples were fixed in 4% paraformaldehyde and processed through gradient ethanol dehydration. The samples were then embedded in paraffin, sectioned, and stained with hematoxylin and eosin (HE). After mounting, the pathological changes in the liver tissue of the mice from each group were observed under a microscope (CX31RTSF, OLYMPUS).

### 2.7. Prussian Blue Staining

The process of tissue embedding and sectioning was performed as described in Section 2.6. For Prussian blue staining, the protocol provided by the manufacturer was strictly followed. After mounting, the liver tissue sections were examined for iron deposition in the livers of the mice across different groups.

### 2.8. Collection of EHW Chemical Constituents

The primary active components of EHW were retrieved from the TCMSP database (https://www.tcmsp-e.com/tcmsp.php (accessed on 3 November 2024)) by searching for “Diercao”. The active ingredients were selected based on the criteria of oral bioavailability (OB) ≥ 30% and drug-likeness (DL) ≥ 0.18.

### 2.9. Screening of Active Ingredient Targets

SMILES identifiers for all compounds of EHW were obtained from the PubChem database (https://pubchem.ncbi.nlm.nih.gov/ (accessed on 3 November 2024)). Potential targets were identified by using the Swiss Target Prediction database (http://www.swisstargetprediction.ch/ (accessed on 3 November 2024)), with targets with a probability greater than 0 considered. Additionally, the Super-PRED database (https://prediction.charite.de/ (accessed on 3 November 2024)) was used to further identify potential targets of EHW active components. The active ingredient targets were then combined with those from the TCMSP database and duplicates were removed, resulting in a comprehensive list of drug targets.

### 2.10. Screening of Liver Injury and Ferroptosis Targets, and Identification of Potential EHW Targets for Liver Injury and Ferroptosis Treatment

Liver-injury-related targets were collected using the GeneCards database (https://www.genecards.org/ (accessed on 18 October 2024)) with the keyword “liver injury,” selecting those with a relevance score > 8.0. Additional liver-injury-related targets were retrieved from the DisGeNET database (https://www.disgenet.org/ (accessed on 2 March 2024)). The two target sets were combined and duplicates removed, forming the disease target set. Ferroptosis-related targets were compiled from the FerrDb database (http://www.zhounan.org/ferrdb (accessed on 27 September 2024)), and duplicates were similarly removed to generate the ferroptosis target set. The three sets of drug active ingredient targets, disease targets, and ferroptosis targets were then intersected using Venn diagrams created by https://www.bioinformatics.com.cn (accessed on 25 December 2024), an online platform to identify potential therapeutic targets for EHW in liver injury through ferroptosis modulation.

### 2.11. Construction of the “Drug–Disease–Active Ingredient–Intersecting Target” Network

The intersecting targets obtained in Section 2.10 were used to construct network and type files, which were then imported into Cytoscape 3.9.1 software to create a “drug–disease–active ingredient–intersecting target” network diagram.

### 2.12. Protein–Protein Interaction (PPI) Network Construction for Intersecting Targets

The intersecting targets identified in Section 2.10 were imported into the String platform (https://string-db.org/ (accessed on 3 November 2024)) to retrieve protein–protein interaction (PPI) data while removing isolated targets. The resulting PPI data were then imported into the Cytoscape 3.9.1 software for topological analysis and visualization.

### 2.13. GO Enrichment and KEGG Pathway Enrichment Analysis

The intersecting targets obtained in Section 2.10 were imported into the DAVID database (https://david.ncifcrf.gov/ (accessed on 3 November 2024)) for Gene Ontology (GO) functional and Kyoto Encyclopedia of Genes and Genomes (KEGG) pathway enrichment analysis. This provided insights into the potential biological processes (BPs), cellular components (CCs), molecular functions (MFs), and signaling pathways involved in the treatment of liver injury through ferroptosis by EHW. Enriched GO functions and KEGG pathways were visualized using bubble charts generated by https://www.bioinformatics.com.cn (accessed on 3 November 2024), an online platform for data analysis and visualization.

### 2.14. Molecular Docking

The SDF format files for the active ingredients of EHW were obtained from the PubChem database. Core protein PDB numbers were determined using Uniprot (https://www.uniprot.org/ (accessed on 5 December 2024)), and the PDB format files for these proteins were downloaded from the PDB database (https://www.rcsb.org/ (accessed on 5 December 2024)). Molecular docking and visualization were performed using the MOE 2022.02 software.

### 2.15. RNA Extraction and Quantitative PCR (qPCR) Analysis

Total RNA was extracted strictly according to the manufacturer’s instructions for the Trizol reagent kit. RNA concentration and quality were assessed using a NanoDrop 200 ultra-micro spectrophotometer. cDNA was synthesized following the guidelines provided by the reverse transcription kit. GAPDH was used as the internal reference gene, and RT-qPCR reactions were performed using a fluorescence quantitative PCR machine (FQD-96X, BIOER). The relative expression of target genes was analyzed using the 2^−ΔΔCt^ method [26]. Primer sequences for the relevant genes were synthesized by Shanghai Sangon Biotech Co., Ltd. (Shanghai, China), with the sequence details provided in Table 1.

### 2.16. Statistical Analysis

Data were analyzed using IBM SPSS Statistics 26, and the results were expressed as the mean ± standard error of the mean (SEM), and statistical significance was determined using one-way ANOVA followed by Duncan’s multiple range test. GraphPad Prism 9 was utilized for data visualization. In all statistical analyses, a *p*-value of less than 0.05 was considered to indicate a statistically significant difference.

## 3. Results

### 3.1. Liver Organ Index and Liver Function Analysis

**Figure 2:**
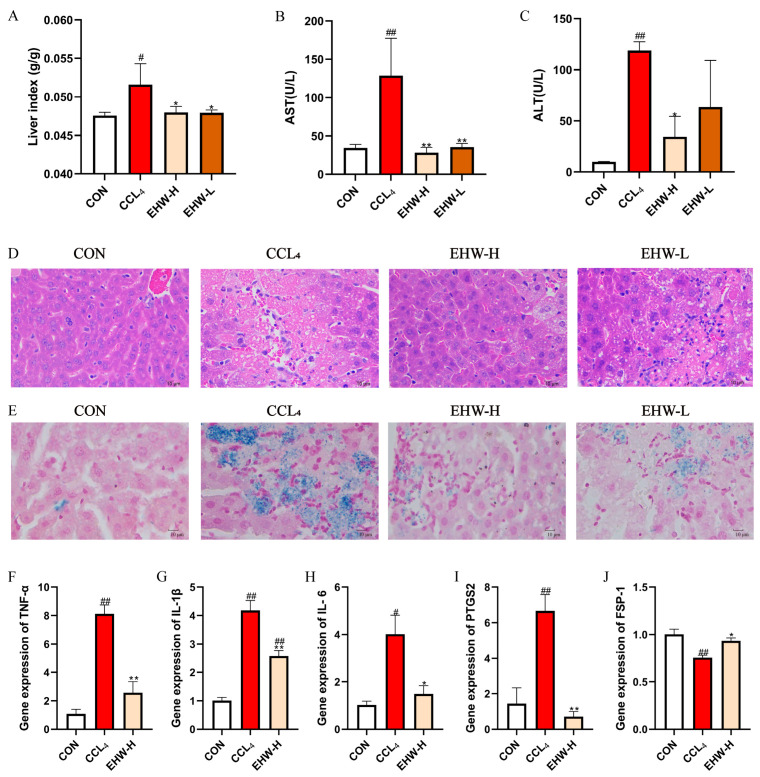
(**A**) Changes in the organ index of mouse livers. (**B**) Changes in serum AST levels. (**C**) Changes in serum ALT levels. (**D**) HE staining results of mouse liver tissue. (**E**) Prussian blue staining results of mouse liver tissue. (**F**) Relative mRNA expression of *TNF-α* in mouse liver. (**G**) Relative mRNA expression of *IL-1β* in mouse liver. (**H**) Relative mRNA expression of *IL-6* in mouse liver. (**I**) Relative mRNA expression of *PTGS2* in mouse liver. (**J**) Relative mRNA expression of *FSP-1* in mouse liver. Note: * indicates a statistically significant difference compared to the CCL_4_ group, # indicates a statistically significant difference compared to the CON group, * *p* < 0.05, ** *p* < 0.01; # *p* < 0.05, ## *p* < 0.01.

The results of the liver organ index for each group of mice are shown in Figure 2A. Compared to the CON group, the liver organ index in the CCL_4_ group was significantly elevated (*p* < 0.05). However, in comparison to the CCL_4_ group, the liver organ index in both the EHW-H and EHW-L groups significantly decreased (*p* < 0.05). The liver function indicators for the EHW treatment groups are presented in Figure 2B,C. Compared to the CON group, serum AST and ALT activities were markedly increased in the CCL_4_ group (*p* < 0.01). Treatment with EHW reversed this trend, as the serum ALT activity in the EHW-H group significantly decreased compared to the CCL_4_ group (*p* < 0.05), and there was a downward trend in the serum ALT activity in the EHW-L group. Additionally, serum AST activity in both the EHW-H and EHW-L groups was significantly reduced (*p* < 0.01), with the EHW-H group showing a more pronounced effect than the EHW-L group.

### 3.2. Pathological Histological Examination of HE Staining

The HE staining results of the liver tissues from the mice in each experimental group are shown in Figure 2D. In the CON group, the liver tissue exhibited a normal structure, with intact hepatocytes and no significant pathological damage or inflammatory cell infiltration. In the CCL_4_ group, hepatic cords were disorganized, and prominent fatty degeneration of the liver was observed, along with extensive hepatocyte necrosis and inflammatory cell infiltration. In contrast, the liver architecture in both the EHW-H and EHW-L groups was partially restored, fatty degeneration was alleviated, hepatocyte necrosis was notably improved, and the number of inflammatory cells infiltrating the liver was significantly reduced. The EHW-H group showed a better therapeutic effect compared to the EHW-L group.

### 3.3. Prussian Blue Staining Examination

The Prussian blue staining results for the liver tissues from each experimental group are shown in Figure 2E. No significant blue iron ferricyanide deposits were observed in the liver tissues of the CON group. In the CCL_4_ group, noticeable blue deposits were present in the areas of liver necrosis, indicating iron overload. In the EHW-H and EHW-L groups, the liver necrosis areas were significantly reduced, and the blue deposition at the necrotic sites was markedly lower than in the CCL_4_ group. Furthermore, the EHW-H group demonstrated a more pronounced reduction in iron deposition compared to the EHW-L group.

### 3.4. Expression of Liver Inflammatory Cytokines and Ferroptosis-Related Genes

The relative expression levels of liver inflammatory cytokines and ferroptosis-related genes in mice are shown in Figure 2F–J. Compared to the CON group, the relative mRNA expression levels of *TNF-α, IL-1β*, and *PTGS2* were significantly elevated in the CCL_4_ group (*p* < 0.01), and *IL-6* mRNA expression was also significantly increased (*p* < 0.05). In contrast, the mRNA levels of *TNF-α, IL-1β,* and *PTGS2* were significantly reduced in the EHW-H group compared to the CCL_4_ group (*p* < 0.01), and *IL-6* expression also decreased significantly (*p* < 0.05). Additionally, the mRNA expression of *FSP-1* was significantly increased in the EHW-H group (*p* < 0.05), while it was significantly decreased in the CCL_4_ group (*p* < 0.01).

### 3.5. Major Active Components of EHW

Based on the criteria of OB ≥ 30% and DL ≥ 0.18 in the TCMSP database, six major active components of EHW were identified. Detailed information on these compounds is presented in Table 2.

### 3.6. Screening of Drug Active Component Targets

Using the TCMSP, Swiss Target Prediction, and SuperPred databases, the targets of the screened active components of EHW were collected and analyzed. After removing duplicates, a total of 578 potential targets for the active components of EHW were obtained.

### 3.7. Potential Targets of EHW for Improving Ferroptosis-Related Liver Injury

**Figure 3:**
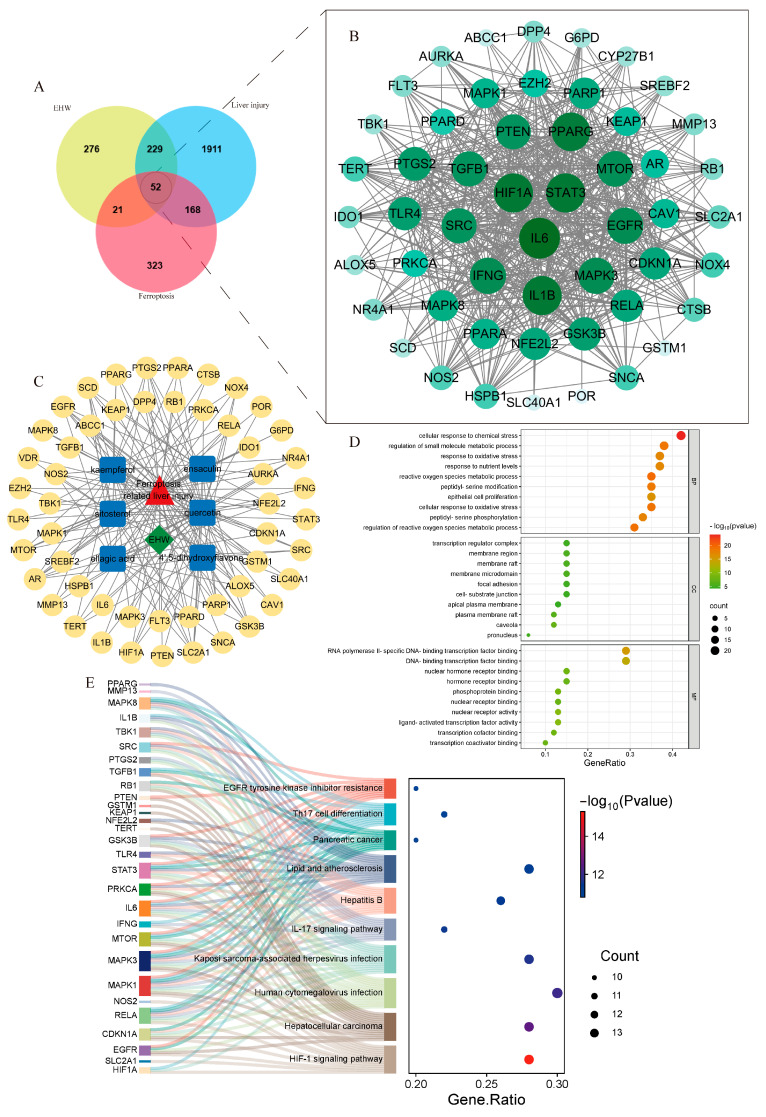
(**A**) Venn diagram of EHW component targets, liver injury targets, and ferroptosis targets. (**B**) PPI network diagram of the intersecting targets. (**C**) “Drug–active component–intersecting target” diagram. (**D**) GO enrichment analysis of EHW’s effects on ferroptosis-related liver injury. (**E**) KEGG enrichment analysis of EHW’s effects on ferroptosis-related liver injury.

By searching the GeneCards database with the keyword “liver injury” and applying the filtering criterion of having a relevance score > 8, the target information was merged with data from the DisGeNET database. After removing duplicate entries, 2360 disease-related targets for liver injury were identified. In the FerrDb database, 564 ferroptosis-related disease targets were obtained. By intersecting the 578 targets of EHW with the 564 ferroptosis-related targets and the 2360 liver injury-related targets, 52 potential targets for EHW in improving ferroptosis-related liver injury were identified. The Venn diagram illustrating these results is shown in Figure 3A.

### 3.8. Construction of PPI Network for the Intersecting Targets

The potential targets of EHW for improving ferroptosis-related liver injury were input into the STRING database to construct a PPI network, and the data were visualized using Cytoscape 3.9.1 software (Figure 3B). By calculating the degree values, the top core targets were found to be *IL-6*, *STAT3*, *HIF1A*, *PTGS2*, *NFE2L2*, and *KEAP1*, among others.

### 3.9. “Drug–Disease–Active Component–Intersecting Target” Network Construction

As shown in Figure 3C, the “drug–disease–active component–intersecting target” relationship network was constructed. The network contained 60 nodes and 184 edges. Based on the degree values, the core active components of EHW for improving ferroptosis-related liver injury were identified. The top-ranked active components included quercetin, kaempferol, and sitosterol.

### 3.10. GO Enrichment Analysis and KEGG Pathway Enrichment Analysis

The intersecting targets were subjected to GO functional enrichment analysis and KEGG pathway enrichment analysis (*p* < 0.05). A total of 2214 BPs, 75 CCs, 132 MFs, and 156 signaling pathways were identified. As shown in Figure 3D,E, the potential targets of EHW for alleviating ferroptosis-related liver injury mainly involved BPs such as response to oxidative stress, the regulation of reactive oxygen species, metabolic processes, and ROS metabolic processes. These targets were predominantly located in cellular components like the transcription regulator complex, membrane regions, and membrane rafts. Molecular functions such as RNA polymerase II-specific DNA-binding transcription factor binding, transcription coactivator binding, and DNA-binding transcription factor binding were the most prominent. The KEGG pathway enrichment results indicated that the enriched pathways were primarily associated with Hepatitis B, the *HIF-1* signaling pathway, and the *IL-17* signaling pathway.

### 3.11. Molecular Docking Results

**Figure 4:**
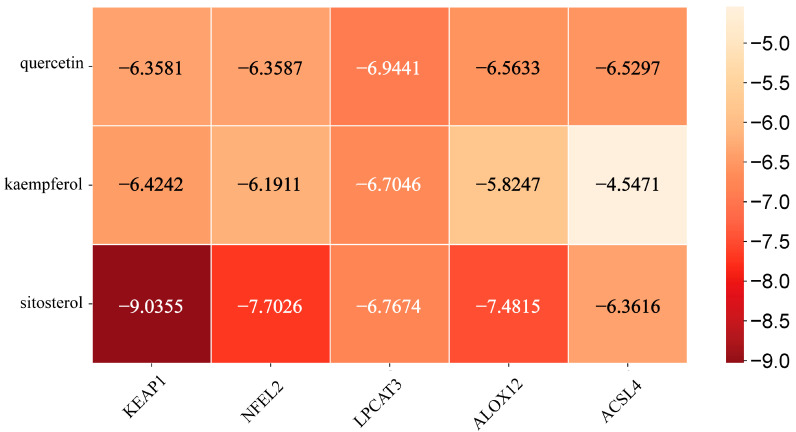
Heatmap of the binding affinity between the key targets related to ferroptosis and liver injury and the active components of EHW.

**Figure 5:**
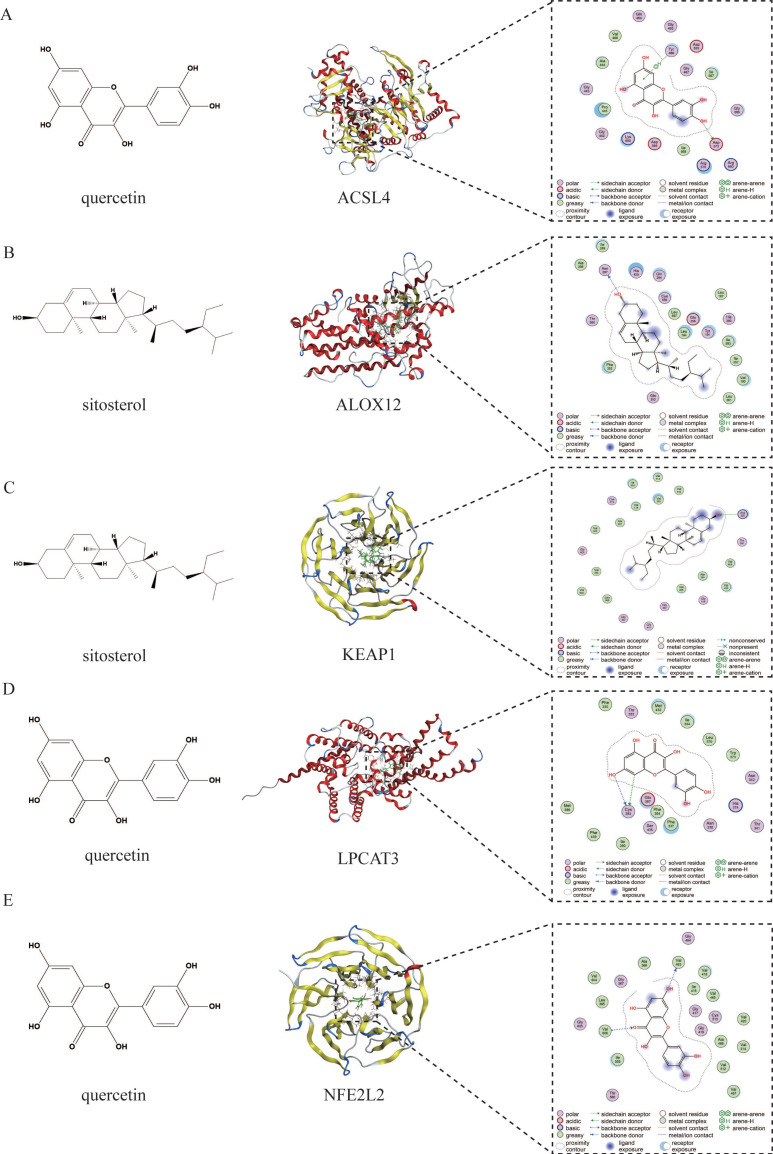
(**A**) Molecular docking visualization of quercetin and *ACSL4*. (**B**) Molecular docking visualization of sitosterol and *ALOX12*. (**C**) Molecular docking visualization of sitosterol and *KEAP1*. (**D**) Molecular docking visualization of quercetin and *LPCAT3*. (**E**) Molecular docking visualization of quercetin and *NFE2L2*.

Figure 4 presents the molecular docking results between the main active components of EHW and the potential key target proteins associated with ferroptosis-related liver injury. The key target proteins identified through topological analysis, including *NFE2L2*, *KEAP1*, *LPCAT3*, *ALOX12*, and *ACSL4*, were pre-processed and subjected to molecular docking alongside the core active components. The results showed that the top three active components, based on their degree value, had binding energies with ferroptosis-related proteins *NFE2L2*, *KEAP1*, *LPCAT3*, *ALOX12*, and *ACSL4* which were lower than −4.5 kcal/mol. Based on the binding energies, the stronger binding component–target combinations were selected for visualization (Figure 5). The results demonstrated that quercetin interacts with the target *ACSL4* at the Tyr468 and ASP573 sites, sitosterol interacts with *ALOX12* at the Asn287 site, sitosterol interacts with *KEAP1* at the Arg326 site, quercetin interacts with *LPCAT3* at the Cys383 site, and quercetin interacts with *NFE2L2* at the Val606 and Val463 sites.

### 3.12. Effect of EHW on the KEAP1-NFE2L2 Signaling Pathway

**Figure 6:**
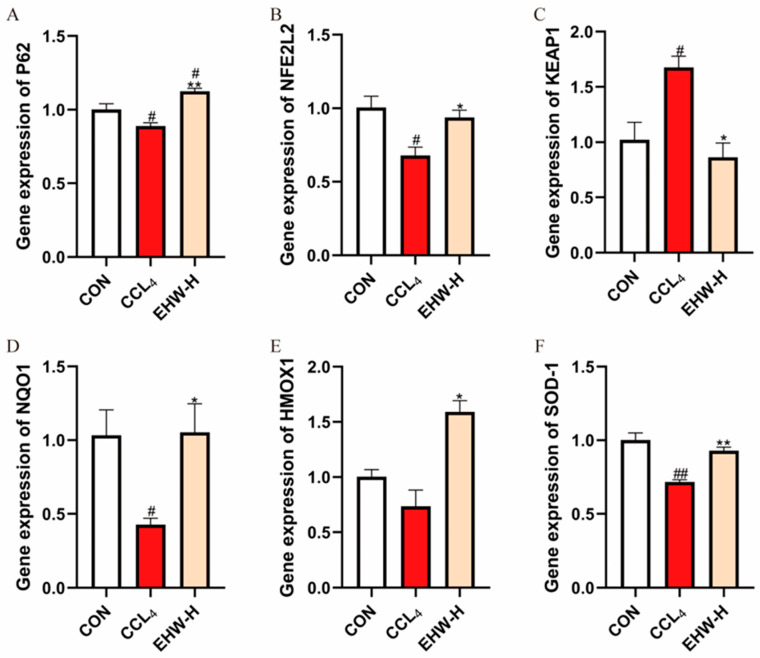
The effect of EHW on the KEAP1-NFE2L2 signaling pathway. (**A**) Relative *P62* mRNA expression. (**B**) Relative *NFE2L2* mRNA expression. (**C**) Relative *KEAP1* mRNA expression. (**D**) Relative *NQO1* mRNA expression. (**E**) Relative *HMOX1* mRNA expression. (**F**) Relative *SOD-1* mRNA expression. Note: * indicates a statistically significant difference compared to the CCL_4_ group, # indicates a statistically significant difference compared to the CON group, * *p* < 0.05, ** *p* < 0.01; # *p* < 0.05, ## *p* < 0.01.

As shown in Figure 6, compared to the CON group, the mRNA expression level of *KEAP1* in the CCL_4_ group was significantly elevated (*p* < 0.05), while the mRNA expression of *SOD-1* was significantly reduced (*p* < 0.01). Additionally, the mRNA expression levels of *P62*, *NFE2L2*, and *NQO1* were significantly decreased (*p* < 0.05), and the mRNA expression level of *HMOX1* showed a downward trend. However, in comparison to the CCL_4_ group, the EHW-H group exhibited a significant reduction in *KEAP1* mRNA expression (*p* < 0.05). Moreover, the mRNA expression of *P62* and *SOD-1* was significantly increased (*p* < 0.01), and the mRNA levels of *NFE2L2*, *NQO1*, and *HMOX1* were all significantly elevated (*p* < 0.05).

### 3.13. Effect of EHW on the Expression of Iron Homeostasis-Related Genes

**Figure 7:**
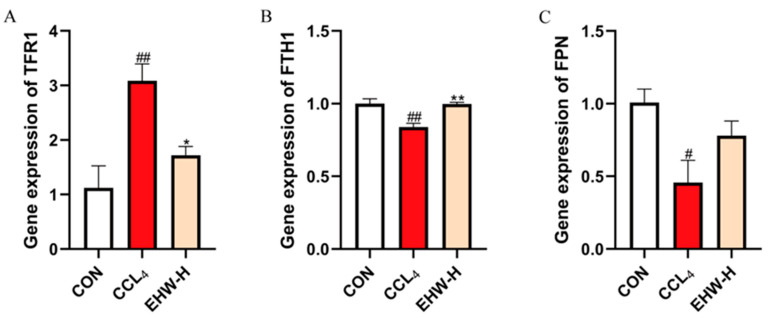
The effect of EHW on iron homeostasis-related genes.(**A**) Relative *TFR1* mRNA expression. (**B**) Relative *FTH1* mRNA expression. (**C**) Relative *FPN* mRNA expression. Note: * indicates a statistically significant difference compared to the CCL_4_ group, # indicates a statistically significant difference compared to the CON group, * *p* < 0.05, ** *p* < 0.01; # *p* < 0.05, ## *p* < 0.01.

As shown in Figure 7, compared to the CON group, the mRNA expression of *TFR1* was significantly elevated in the CCL_4_ group (*p* < 0.01), while the mRNA expression of *FTH1* was significantly reduced (*p* < 0.01). Additionally, the mRNA expression level of *FPN* was significantly decreased (*p* < 0.05). In contrast, when compared to the CCL_4_ group, the EHW-H group showed a significant reduction in *TFR1* mRNA expression (*p* < 0.05), while *FTH1*’s mRNA expression was significantly increased (*p* < 0.01), and the mRNA expression of *FPN* exhibited an upward trend.

### 3.14. Effect of EHW on the Expression of Genes in the PLOOH Metabolism Pathway

**Figure 8:**
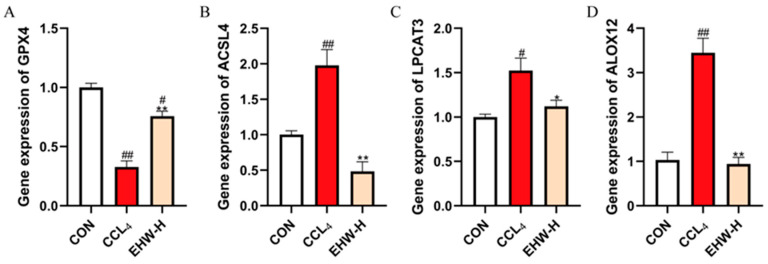
The effect of EHW on gene expression in the PLOOH metabolism pathway. (**A**) Relative *GPX4* mRNA expression. (**B**) Relative *ACSL4* mRNA expression. (**C**) Relative *LPCAT3* mRNA expression. (**D**) Relative *ALOX12* mRNA expression. Note: * indicates a statistically significant difference compared to the CCL_4_ group, # indicates a statistically significant difference compared to the CON group, * *p* < 0.05, ** *p* < 0.01; # *p* < 0.05, ## *p* < 0.01.

As shown in Figure 8, compared to the CON group, the mRNA expression levels of *ACSL4* and *ALOX12* were significantly increased in the CCL_4_ group (*p* < 0.01), and the mRNA expression of *LPCAT3* was also significantly elevated (*p* < 0.05). On the other hand, the mRNA expression of *GPX4* was significantly decreased (*p* < 0.01). In contrast, when compared to the CCL_4_ group, the EHW-H group exhibited a significant reduction in the mRNA expression of *ACSL4* and *ALOX12* (*p* < 0.01), as well as a decrease in the mRNA expression of *LPCAT3* (*p* < 0.05). Moreover, the mRNA expression of *GPX4* was significantly increased in the EHW-H group (*p* < 0.01).

## 4. Discussion

Liver injury is a common condition in veterinary clinical practice and poses a significant threat to livestock production [27]. Inhibiting excessive hepatocyte death is an effective approach to protect the liver and treat liver diseases [28]. Increasing evidence suggests that ferroptosis plays a crucial role in the occurrence and progression of liver-injury-related diseases [29,30]. Fortunately, with the rapid development of traditional Chinese (veterinary) medicine research, its potential use in liver injury treatment demonstrating safety, reliability, and multi-target effects has garnered increasing attention [31]. In this study, we established a mouse model of liver injury using CCL_4_, a classic hepatotoxic substance. The results showed that, compared to the CON group, the CCL_4_ group had a significantly increased liver index, and serum AST and ALT activities were markedly elevated. Histological examination via HE staining revealed disorganized hepatic cords, hepatocyte fatty degeneration, and considerable hepatocyte necrosis with inflammatory cell infiltration in the CCL_4_ group. However, EHW successfully reversed these trends. Compared to the CCL_4_ group, both the EHW-H and EHW-L groups exhibited a significant reduction in the liver index, and serum AST activity was markedly decreased. Serum ALT activity in the EHW-L group showed a decreasing trend. Consistently, in the EHW-H group, serum ALT activity significantly decreased. HE staining also revealed that liver architecture was restored in both the EHW-H and EHW-L groups, hepatocyte fatty degeneration was alleviated, hepatocyte necrosis was notably improved, and the area of inflammatory cell infiltration was substantially reduced, with the EHW-H group showing a better therapeutic effect than the EHW-L group. The excessive production and release of pro-inflammatory cytokines is one of the major characteristics of liver injury, and *TNF-α* generation is considered an important early marker of CCL_4_-induced liver injury [32]. In this study, compared to the CON group, the relative mRNA expression levels of *TNF-α* and *IL-1β* in the CCL_4_ group were significantly increased, and *IL-6* mRNA expression was also significantly elevated. In contrast, compared to the CCL_4_ group, the EHW-H group showed a significant decrease in the relative mRNA expression levels of *TNF-α* and *IL-1β*, and *IL-6* mRNA expression was significantly reduced. These results suggest that EHW has a protective effect against CCL_4_-induced liver injury, and this protective effect appears to be dose-dependent.

Ferroptosis is an iron-dependent form of programmed cell death, and abnormal iron accumulation is a key feature in the onset of ferroptosis [33]. *PTGS2* is considered an important marker of ferroptosis [34], and *FSP-1* is a recently identified inhibitor that plays a crucial role in suppressing ferroptosis [35]. To further investigate whether the hepatoprotective effect of EHW is related to the alleviation of ferroptosis in hepatocytes, we first performed Prussian blue staining. The results revealed that in the CCL_4_ group, there was a significant increase in blue precipitates in the areas of liver necrosis, indicating the presence of iron overload. In contrast, in the EHW treatment groups, the areas of liver necrosis were significantly reduced, and the extent of blue precipitate staining in the liver tissue was markedly less than in the CCL_4_ group. Furthermore, we measured the relative mRNA expression levels of *PTGS2* and *FSP-1* in the liver. Compared to the CON group, the relative mRNA expression of *PTGS2* was significantly elevated in the CCL_4_ group, while the expression of *FSP-1* was significantly reduced. In contrast, in the EHW-H group, the relative mRNA expression of *PTGS2* was significantly decreased, while the expression of *FSP-1* was notably increased compared to the CCL_4_ group. These results suggest that CCL_4_-induced liver injury is closely associated with the excessive occurrence of ferroptosis, and that EHW may alleviate ferroptosis, thereby exerting its hepatoprotective effects.

Network pharmacology, an interdisciplinary research approach that integrates knowledge from fields such as systems biology and pharmacology, enables the analysis of the systemic effects of multi-component drugs on the human body [36]. It has significant advantages in exploring the therapeutic targets of TCM and investigating disease mechanisms [37,38]. In this study, by constructing an intersecting target PPI network, we identified the core targets with higher degree values, including *PTGS2*, *NFE2L2*, *KEAP1*, and others. These targets exhibited frequent interactions with the major active compounds of EHW, such as quercetin, kaempferol, and sitosterol, suggesting that EHW may exert its hepatoprotective effects by targeting these core molecules. GO enrichment analysis, which linked the genes identified in this study to functional categories in the Gene Ontology database, revealed statistically significant differences in specific biological processes, cellular components, and molecular functions [39]. KEGG analysis aids in understanding the regulatory processes of various metabolic pathways and cellular processes in biological systems, thus shedding light on key biological mechanisms and interactions [40]. In this study, GO enrichment analysis indicated that the potential targets of EHW in alleviating ferroptosis-induced liver injury were mainly involved in BPs such as the response to oxidative stress, regulation of reactive oxygen species (ROS) metabolic processes, and ROS metabolic processes. These targets were predominantly localized in cellular components, including the transcription regulator complex, membrane regions, and membrane rafts. In terms of molecular functions, they were primarily associated with RNA polymerase II-specific DNA-binding transcription factor binding, transcription coactivator binding, and DNA-binding transcription factor binding. KEGG pathway enrichment analysis revealed that the enriched pathways were primarily related to the Hepatitis B, *HIF-1* signaling, and *IL-17* signaling pathways. Molecular docking is a computational biology method typically used to study the interactions between drugs and their targets. In this study, molecular docking of the three top-ranking active compounds with key ferroptosis targets (*KEAP1*, *NFE2L2*, *LPCAT3*, *ALOX12*, and *ACSL4*) demonstrated that the binding energies of the target small molecule complexes were all significantly lower than −4.5 kcal/mol, suggesting a strong affinity between the active components and the targets. The visualization results further indicated that the active components and core targets were closely connected in a specific spatial structure, implying that the major active compounds of EHW have a strong biological affinity with the ferroptosis-related core targets. These findings suggest that EHW may exert its hepatoprotective effects by acting on the KEAP1-NFE2L2 signaling pathway and reducing the accumulation of PLOOH, thereby mitigating hepatocyte ferroptosis.

Ferroptosis is a form of cell death closely associated with oxidative stress [41]. The KEAP1-NFE2L2 pathway is a crucial regulator of cellular oxidative stress [42]. Upon activation, *NFE2L2* exerts potent antioxidant effects and plays a key role in the regulation of ferroptosis [43]. *KEAP1* negatively regulates *NFE2L2* [44], while *P62* can interact with *KEAP1* at the *NFE2L2* binding site, competing with *KEAP1* and thereby activating *NFE2L2* [45]. Once activated, *NFE2L2* induces an increase in *P62*, creating a positive feedback loop [46]. More importantly, when *NFE2L2* is activated, the downstream antioxidant genes such as *HMOX1*, *NQO2*, and *SOD-1* are activated, directly exerting antioxidant cell protection and alleviating ferroptosis [47]. In this study, compared to the CON group, the CCL_4_ group showed a significant increase in *KEAP1* mRNA expression, a marked decrease in *SOD-1* mRNA expression, and significant reductions in the mRNA expression levels of *P62*, *NFE2L2*, and *NQO1*, with *HMOX1* mRNA expression also showing a decreasing trend. In contrast, compared to the CCL_4_ group, the EHW-H group showed a significant decrease in *KEAP1* mRNA expression, a dramatic increase in *P62* and *SOD-1* mRNA expression, and significant increases in the mRNA expression levels of *NFE2L2*, *NQO1*, and *HMOX1*. These results suggest that EHW can activate *NFE2L2*, upregulate the expression of antioxidant genes, reduce cellular oxidative stress and ferroptosis, and thereby exert a protective effect against liver injury.

Studies have shown that the dysregulation of iron metabolism is a critical trigger for ferroptosis [48]. The excessive accumulation of iron in the labile iron pool (LIP) is a key factor in the initiation of ferroptosis [49]. The iron content in the LIP is tightly regulated by various molecules. Under normal physiological conditions, iron ions typically interact with transferrin, after which they are internalized into the cell via *TFR1* [50]. Once inside the cell, iron is either stored in the iron storage protein complex or, alternatively, exported by *FPN* [51,52]. Notably, *FTH1* serves as a critical component of the iron storage protein complex [53]. When *TFR1* is overexpressed [54], *FTH1* is insufficient [55] or *FPN* is suppressed [56], and iron accumulates abnormally in the LIP, leading to the Fenton reaction, which triggers lipid peroxidation and ultimately induces ferroptosis [57]. Interestingly, recent studies have discovered that the activation of the KEAP1-NFE2L2 pathway not only regulates the body’s antioxidant response but also plays a critical role in iron metabolism. Numerous studies have confirmed that key regulators of iron metabolism, such as *FTH1* and *FPN*, are controlled by *NFE2L2* [58]. In this study, compared to the CON group, the CCL_4_ group showed a significant increase in *TFR1* mRNA expression, a marked decrease in *FTH1* mRNA expression, and a significant reduction in *FPN* mRNA expression. In contrast, the EHW-H group exhibited a significant decrease in *TFR1* mRNA expression, a dramatic increase in *FTH1* mRNA expression, and an upward trend in *FPN* mRNA expression compared to the CCL_4_ group. These results suggest that EHW may regulate iron metabolism by activating the KEAP1-NFE2L2 pathway, restoring cellular iron homeostasis, and inhibiting excessive ferroptosis, thereby alleviating liver injury.

The accumulation of phospholipid hydroperoxides (PLOOHs) is the most direct cause of ferroptosis [59]. Timely clearance of PLOOHs or the inhibition of the synthesis of their precursors, phospholipid-containing polyunsaturated fatty acid chains (PUFA-PL), can exert a positive effect on ferroptosis [60]. *GPX4* is the most well-known reductase of PLOOHs, as it reduces PLOOHs to their corresponding alcohols [34]. Notably, the expression of this gene is also regulated by *NFE2L2* [61]. Another critical pathway to prevent ferroptosis is the inhibition of PUFA-PL synthesis. *ACSL4* is a fatty acid synthetase that specifically catalyzes the activation of long-chain polyunsaturated fatty acids (PUFAs) [62]. Under the catalysis of *ACSL4*, these long-chain PUFAs are conjugated with coenzyme A (CoA) to form PUFA-CoA intermediates [63]. Subsequently, *LPCAT3* esterifies these intermediates to generate PUFA-PL [64], which is ultimately converted into PLOOHs through the action of lipoxygenases such as *ALOX12* [65]. In this study, compared to the CON group, the CCL_4_ group showed a dramatic increase in the mRNA expression of *ACSL4* and *ALOX12*, a significant increase in *LPCAT3* mRNA expression, and a marked decrease in *GPX4* mRNA expression. In contrast, the EHW-H group exhibited a significant reduction in the mRNA expression of *ACSL4* and *ALOX12*, a notable decrease in *LPCAT3* mRNA expression, and a dramatic increase in *GPX4* mRNA expression compared to the CCL_4_ group. These results suggest that EHW may reduce the generation of PLOOHs and accelerate their reduction, thereby decreasing the accumulation of PLOOHs, inhibiting ferroptosis, and alleviating liver injury.

## 5. Conclusions

In summary, EHW may exert its hepatoprotective effects by regulating the KEAP1-NFE2L2 signaling pathway to alleviate oxidative stress, restore cellular iron homeostasis, reduce the accumulation of PLOOHs, and ultimately mitigate ferroptosis in the liver. On the other hand, despite promising results, the direct applicability of EHW in veterinary practice requires further pharmacokinetic, toxicity, and large-scale clinical trials to confirm its safety and effectiveness across different livestock breeds.

## Figures and Tables

**Table 1:** Sequences of primers for RT-qPCR.

Gene	Primer (5′→3′)	Length (bp)
*IL-1β*	F: CACTACAGGCTCCGAGATGAACAAC R: TGTCGTTGCTTGGTTCTCCTTGTAC	145
*IL-6*	F: CTTCTTGGGACTGATGCTGGTGAC R: TCTGTTGGGAGTGGTATCCTCTGTG	91
*TNF-α*	F: CGCTCTTCTGTCTACTGAACTTCGG R: GTGGTTTGTGAGTGTGAGGGTCTG	113
*PTGS2*	F: TCTGGTGCCTGGTCTGATGATG R: CTATGAGTATGAGTCTGCTGGTTTGG	134
*FSP-1*	F: AGAACCGGATGGTGTTGCTAC R: CACCTCGTTAAACTTGCCAGG	104
*TFR1*	F: AGCCAGATCAGCATTCTCTAACTTG R: CTCCACATGACTGTTATCTCCATCTAC	100
*FTH1*	F: CAAGTGCGCCAGAACTACCA R: GCCACATCATCTCGGTCAAAA	122
*FPN*	F: ACCAAGGCAAGAGATCAAACC R: AGACACTGCAAAGTGCCACAT	138
*GPX4*	F: CTCCGAGTTCCTGGGCTTGTG R: CCGTCGATGTCCTTGGCTGAG	87
*ACSL4*	F: CTCACCATTATATTGCTGCCTGT R: TCTCTTTTTGCCATAGCGTTTTTCT	116
*LPCAT3*	F: CCCCACATCACAGACGACTATC R: TCTCACGGTCCCATTTTCATC	192
*ALOX12*	F: ACCTCAGACAATAGCAGCGGA R: TCAACGTCCATTCAAAGTCCAG	92
*P62*	F: GAACTCGCTATAAGTGCAGTGT R: AGAGAAGCTATCAGAGAGGTGG	131
*NFE2L2*	F: GTTGCCACCGCCAGGACTAC R: AAACTTGTACCGCCTCGTCTGG	88
*KEAP1*	F: CTGGAGGATCATACCAAGCAGG R: GGATACCCTCAATGGACACCAC	220
*NQO1*	F: TGGCCGAACACAAGAAGCTG R: GCTACGAGCACTCTCTCAAACC	112
*HMOX1*	F: CTGGAGATGACACCTGAGGTCAA R: CTGACGAAGTGACGCCATCTG	150
*SOD1*	F: GGAACCATCCACTTCGAGCAG R: ACAGCCTTGTGTATTGTCCCC	126
*GAPDH*	F: GTCGTGGAGTCTACTGGTGTCTTC R: AGTTGTCATATTTCTCGTGGTTCA	145

**Table 2:** Information on the major active components of EHW.

Molid	Active Ingredient	OB (%)	DL
MOL001002	ellagic acid	43.06	0.43
MOL000359	sitosterol	36.91	0.75
MOL000422	kaempferol	41.88	0.24
MOL006326	ensaculin	45.76	0.86
MOL006331	4′,5-dihydroxyflavone	48.55	0.19
MOL000098	quercetin	46.43	0.28

## Data Availability

The data presented in this study are available on request from the corresponding author.

## Abbreviations

The following abbreviations are used in this manuscript:

EHW	*Euphorbia humifusa* Willd. ex Schltdl.
TCM	Traditional Chinese Medicine
AST	Aspartate aminotransferase
ALT	Alanine aminotransferase
HE	Hematoxylin–eosin staining
OB	Oral bioavailability
DL	Drug-likeness
PPI	Protein–protein interaction
GO	Gene Ontology
KEGG	Kyoto Encyclopedia of Genes and Genomes
BP	Biological process
CC	Cellular component
MF	Molecular function
SEM	Standard error of the mean
LIP	Labile iron pool
PLOOHs	Phospholipid hydroperoxides
PUFA-PL	Phospholipid-containing polyunsaturated fatty acid chains
PUFAs	Polyunsaturated fatty acids
CoA	Coenzyme A
